# N-Terminal-Based Targeted, Inducible Protein Degradation in *Escherichia coli*

**DOI:** 10.1371/journal.pone.0149746

**Published:** 2016-02-22

**Authors:** Karthik Sekar, Andrew M. Gentile, John W. Bostick, Keith E. J. Tyo

**Affiliations:** 1 Department of Chemical and Biological Engineering, Northwestern University, Evanston, IL, United States of America; 2 Master of Biotechnology Program, Northwestern University, Evanston, IL, United States of America; 3 Department of Pathology, Northwestern University, Chicago, IL, United States of America; 4 Department of Microbiology-Immunology, Northwestern University, Chicago, IL, United States of America; Baylor College of Medicine, UNITED STATES

## Abstract

Dynamically altering protein concentration is a central activity in synthetic biology. While many tools are available to modulate protein concentration by altering protein synthesis rate, methods for decreasing protein concentration by inactivation or degradation rate are just being realized. Altering protein synthesis rates can quickly increase the concentration of a protein but not decrease, as residual protein will remain for a while. Inducible, targeted protein degradation is an attractive option and some tools have been introduced for higher organisms and bacteria. Current bacterial tools rely on C-terminal fusions, so we have developed an N-terminal fusion (Ntag) strategy to increase the possible proteins that can be targeted. We demonstrate Ntag dependent degradation of mCherry and beta-galactosidase and reconfigure the Ntag system to perform dynamic, exogenously inducible degradation of a targeted protein and complement protein depletion by traditional synthesis repression. Model driven analysis that focused on rates, rather than concentrations, was critical to understanding and engineering the system. We expect this tool and our model to enable inducible protein degradation use particularly in metabolic engineering, biological study of essential proteins, and protein circuits.

## Introduction

A common modality in synthetic biology is to modulate the abundance of proteins for catalysis [1], computation [2], detection [3], and programmed therapeutics [4]. The vast majority of tools to modulate protein activity change mRNA and protein synthesis rates [5]. Such tools include transcriptional repressors [6, 7], RNAi [8], riboregulators [9], and more recent tools using catalytically-inactivated Cas9, dCas9 [10].

Tools that change synthesis rates excel in applications where rapid transition to an ON state is required, specifically the increase in concentration of a desired protein, as transcription and translation occur on a time scale of minutes [11, 12]. Turning OFF proteins (i.e. decreasing concentration) is indirect using synthesis tools. Protein degradation and dilution-by-growth reduce protein concentration when synthesis is low. In practice, most native proteins have long half-lives (over 20 hours) [13–15]; therefore, dilution is the primary mechanism to deplete these proteins. Dilution is directly related to growth rate; therefore, turning off the protein slows as growth slows [16]. This complicates knockdown of essential proteins because lower essential protein concentrations likely results in a slowed growth phenotype, where dilution and reducing the essential protein concentration is even further slowed.

Therefore to rapidly generate OFF states, degradation is much more desirable. Genetically-encoded targeted and inducible protein degradation is an attractive solution and has been demonstrated in a few strategies. A target protein is genetically fused to a destabilization tag. In prokaryotes, the tagged protein is stable, until degradation is induced by expression of a protease or adaptor protein, resulting in rapid decrease in protein concentration [17–19]. Several demonstrations of targeted, exogenous protein degradation have been applied recently for metabolic engineering [20, 21], fundamental study of essential proteins [22]**,** enhancing recombinant protein production [23]**,** and protein-based circuits [24]. Most of these studies target essential proteins that otherwise could not be genetically knocked out.

Induced protein degradation strategies require that prior to induction the tag does not destabilize the protein (i), significantly affect native function (ii), or cause the tag to be inaccessible to the adaptor or protease (iii). Ideally, tagging can be done on the N- or C-terminus; however, all current tools for bacteria are limited to C-terminus [17–19]. This reduces the set of proteins able to be knocked down. For example, the mazE antitoxin is an essential protein [25] in *Escherichia coli* and requires a free C-terminus for native activity [26]. This protein may not be able to be tagged on its C-terminus and consequently may not be able to be studied using state-of-the-art induced protein degradation methods.

**Fig 1:**
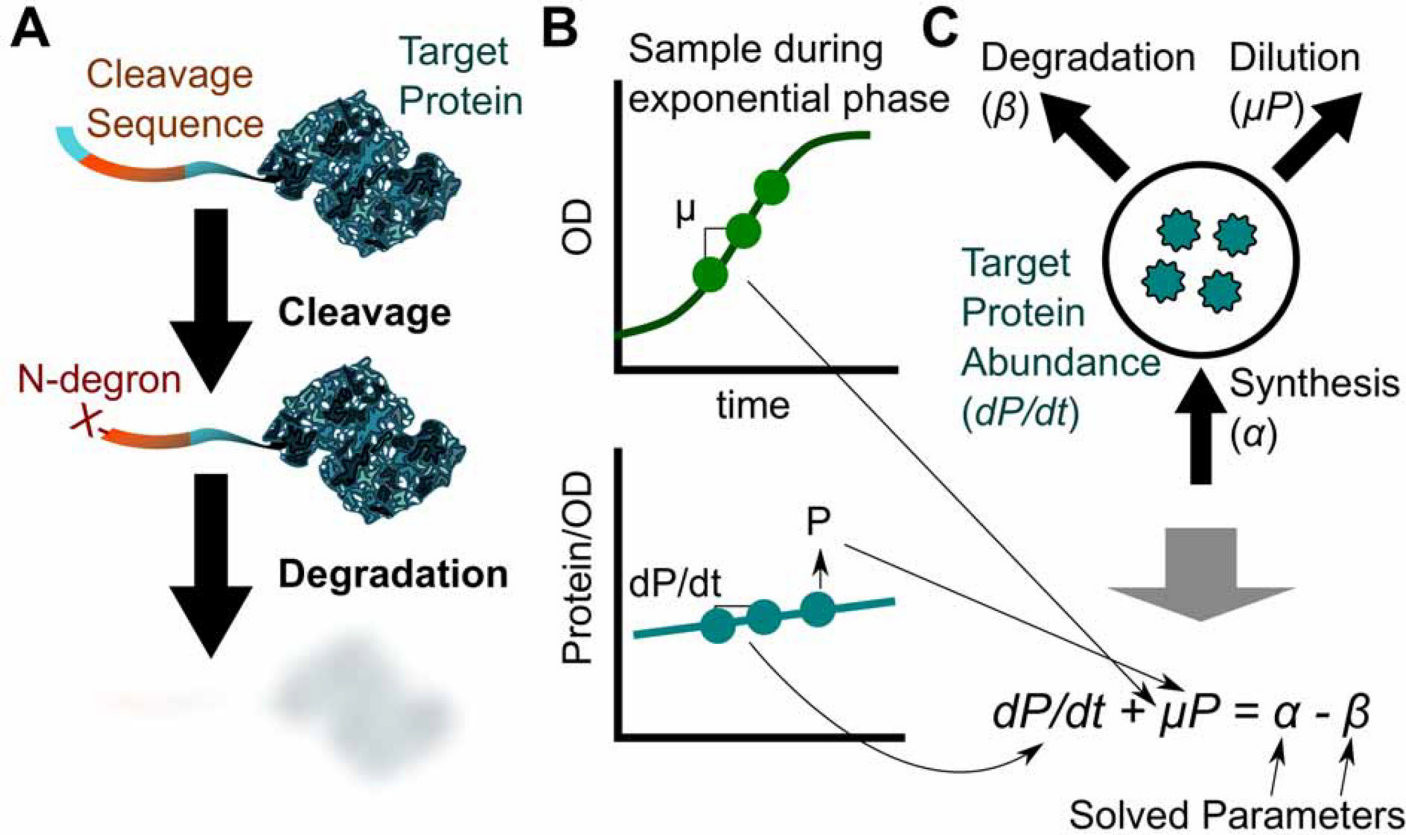
Targeted degradation is achievable using N-terminal fusions and elucidated with model-driven analysis. (**A**) A cleavable tag is fused N-terminally to the target protein. On cleavage, an N-degron is exposed leading to degradation of the target. (**B**) Balanced exponential phase growth experiments can be used to estimate parameters for a quantitative model (**C**) to calculate degradation rates. See **S5 Fig** for a detailed demonstration of calculations.

In response, we designed and characterized an N-degron tag (Ntag) for *E*. *coli* based loosely on the TEV-Induced Protein Instability/Inactivation (TIPI) system previously developed in *Saccharomyces cerevisae* [27] (**Fig 1A**). In this system, the target protein is N-terminally modified with a protection domain that hides an N-degron (an amino acid that destabilizes a protein when it is on the extreme N-terminus). When an encoded protease site is cleaved, the N-degron (non-methionine amino acid) is exposed. The protein is then degraded through the host N-end rule pathway. As we will discuss later, we do not use TEV for induction, but instead rely on expression of N-end rule pathway proteins, which in bacteria utilizes the ClpAP protease complex and the adaptor protein ClpS [28–30].

The degradome of prokaryotes is used to regulate growth rates, convoluting the effects of degradation and dilution-by-growth [31, 32]. We utilize a model-driven framework to characterize the degradation rates in *E*. *coli*. Our analysis simultaneously accounted for changes in protein abundance related to targeted degradation, synthesis, and dilution in balanced, exponentially growing cultures (**Fig 1B and 1C**). For example, if degradation and dilution are increased, but not greater than synthesis, the protein abundance would still increase over time, masking the increased degradation.

In this study, we evaluated the genetic conditions that affect degradation rate toward optimizing the Ntag degradation system. We studied changes in degradation rate during balanced exponential growth. Growth and protein abundance were measured over time and fit to a differential equation. We confirm essential proteins (ClpP, ClpA, ClpS, and ClpX) in the degradation pathway support high degradation rates, and use these to demonstrate a dynamic induction of protein degradation via temporal availability of ClpP. We expect our Ntag to expand the applications of inducible protein degradation to metabolic engineering, protein circuit, and biological investigations.

## Materials and Methods

### Bacterial Strains and Media

All *E*. *coli* strains used in this study are described in **Table A in S1 File**. DH5α and Top10 strains were used for all plasmid construction. Derivatives of the MG1655 Z1 strain were used for all experiments [33]. The MG1655 Z1 strain constitutively expresses TetR and LacI so that synthesis of P_Ltet-O_ and P_trc_ promoters can be controlled through titration of doxycycline and IPTG respectively. Knockouts were introduced (*malE*, *sspB*, *clpASXP*, *aat*) to the Z1 strain using p1vir phage transduction from Keio library strains [25] (from Yale University *E*. *coli* Genetic Stock Center) and knockouts were verified by colony PCR. All kanamycin markers were excised using the pCP20 plasmid [34] using a previously described protocol [35].

Luria-Bertani (LB) media (10 g/L of tryptone, 5 g/L of yeast extract, 10 g/L of NaCl) with 0.4% glucose added to prevent carbon limitation [36] was used for all experiments. 40 ng/mL doxycycline was used to maintain expression of reporter proteins (Ntag-RFP, RFP, and Ntag-beta-galactosidase) and 17 mg/L chloramphenicol to maintain the plasmid. In experiments using pTrc-based plasmids, 100 mg/L of ampicillin was added to maintain the plasmid and 1 mM of IPTG was used to express the inserts.

### Plasmid construction

All plasmids used in this study are listed and described in **Table B in S1 File**. All plasmids were constructed using Gibson Assembly techniques [37] and designed using the j5 software suite [38]. Construction oligonucleotides are listed in **Table C in S1 File**, and checking oligonucleotides are listed in **Table D in S1 File**. The plasmids which expressed the N-tagged proteins (called target plasmid from here on) were all pSC101-derived (specifically BioBricks pSB4C5). The Ntag sequence was codon optimized for *E*. *coli* and synthesized chemically (IDT, San Jose, CA). Details of the Ntag sequence and its motifs are provided in **S1 File.** Protein expression was driven by a P_Ltet-O_ promoter and titratable using doxycycline (**S1 Fig**). A second plasmid bearing the proteins to modulate degradation (called the perturbation plasmid from here on) were all derived from pTrcHis2B. All plasmids were sequence verified and are available from AddGene (Deposit # 71910). All GenBank files are included in **S2 File**.

### Culture Conditions

For all culture experiments, cells were freshly transformed with plasmids the previous day. Colonies were picked and cultured. Once concentration exceeded OD_600_ 1.0, cells were inoculated into 250 mL baffled shake flasks (working volume of 25 mL) at 1:1000 dilution. Shake flasks, once inoculated, were kept cold (12°C) until the next day and then cultivated at 37°C at 250 RPM. Sampling began once exponential phase was established (4–5 doublings, usually 2–5 hours from start of shaking) and measurements of protein activity and OD_600_ occurred at least once an hour. OD was measured with UV-1800 Spectrophotometer (Shimadzu, Kyoto, Japan). Cultures that reached OD values beyond 1 were diluted into fresh shake flasks with prewarmed media of the same constituents to minimize transition disturbances and keep the cultures under balanced growth for continued measurements. All raw data for culture experiments are available in **S3 and S4 Files**.

### Cleavage Assay

Cultures with relevant plasmids and inducers were grown to stationary phase (OD 5–10) in the presence of 100 ng/mL of anhydrotetracycline to maintain maximal synthesis of MBP-Ntag-RFP (compared to doxycycline). Cultured cells were then pelleted and resuspended in cold Tris-HCl buffer (50 mM, pH 7.5) with 1 mg/ml lysozyme. The samples were then incubated on ice for 10 minutes and frozen with dry ice/ethanol. Frozen cultures were then thawed on ice and centrifuged to separate protein from cellular debris at 17,000 g for 10 minutes in a precooled centrifuge. A Bradford assay (Biorad, Berkeley, CA) was used to quantify protein content in the lysate. Samples were diluted to 10 μg/mL with 0.1x Sample Buffer supplied with Wes Rabbit Kits (ProteinSimple, San Jose, CA). A polyclonal rabbit anti-FLAG primary antibody (#ab1162, Abcam, Cambridge, UK; Antibody Registry: AB_298215) and a monoclonal mouse MBP-HRP conjugated antibody (#e8038, New England Biolabs, Ipswitch, MA; Antibody Registry: AB_1559732) were used to visualize the bands in the Wes machine (ProteinSimple, San Jose, CA) using the standard instrument protocol. Both antibodies were used at a dilution of 1:50 (as recommended by the protocol) with the supplied Wes Antibody Diluent solution.

### Fluorescence Assay

For RFP fluorescence measurements, 200 μL of culture was immediately chilled on ice and then measured (λ_ex_ = 585 nm; λ_em_ = 615 nm) on a Synergy H1 Plate Reader (Biotek, Winooski, VT) at a gain of 100. A non-RFP producing *E*. *coli* culture in LB media sample was used as the blank for the fluorescence measurement.

### Beta-galactosidase assay

For beta-galactosidase optical density measurements, 0.5 mL of culture was placed into cold microcentrifuge tubes kept on ice. Cultures were pelleted at 5,000 g for 5 minutes using a precooled centrifuge. Cells were then resuspended to an OD of approximately 1 with PBS solution. 25 μL of concentrated cells were added to 100 μL permeabilization solution (100 mM Na_2_HPO_4_, 20 mM KCl, 2 mM MgSO_4_, 0.8 mg/mL CTAB (hexadecyltrimethylammonium bromide), 0.4 mg/mL sodium deoxycholate, 10 mg/mL bovine serum albumin, and 5.4 μL/mL beta-mercaptoethanol. Permeabilization solution was always made fresh on the day of assay from stock solutions. Standards were also prepared from purified *E*. *coli* beta-galactosidase (Sigma Aldrich, St. Louis, MO) in PBS and added to permeabilization solution in same manner as cells. After the last sample was collected for the day, all samples (cells and perm solution mix) were removed from room temperature and incubated at 30°C for at least 30 minutes. 90 μL of prewarmed (30°C) substrate solution (60 mM Na_2_HPO_4_, 40 mM NaH_2_PO_4_, 1 mg/mL o-nitrophenyl-β-D-Galactoside (ONPG), and 2.7 μL/mL β-mercaptoethanol) was dispensed into wells on a flat-bottom 96 well plate. 15 μL of each sample was added to designated wells, mixed for a few minutes using a platform shaker, and absorbance at 420 nm was read over one hour every five minutes in Synergy H1 plate reader. Plates were sealed with plastic film and kept warm (30°C) in plate reader during reading. Activity was measured as the change in OD over time, using the standards to calibrate to specific activity. All samples were measured in technical replicate.

### Culture conditions for induction of ClpAP pathway proteins

For testing the effects of inducing ClpAP pathway proteins, cultures were first grown in the pre-induction condition in shake flasks (LB + 0.4% glucose, 40 ng/mL doxycycline, no IPTG), where the target protein (mCherry) was expressed. Upon OD reaching between 0.1 and 0.3, 1 mL of culture was sampled, centrifuged at 5,000 g for 5 minutes, and resuspended with warmed (37°C) PBS. Samples were centrifuged again in the same conditions and then inoculated into a fresh shake flask with new media. For “Repression Only” experiment, the plasmids pKS012 (Ntag-RFP) and pTrc (empty plasmid) were used and the post-induction media had no doxycycline but had IPTG to control for cellular effects from IPTG. For the “Degradation and Repression” condition, the pKS012 (Ntag-RFP) and pKS044 (ClpP expression) plasmids were used and the post-induction media had IPTG but no doxycycline. The fluorescence and OD at the zero time point was determined by extrapolating a negative control condition (no knockdown initiated) back to t = 0 h. All raw data for induction experiments are available in **S6 File**.

### Model specifications and analysis

Change in protein abundance is related to synthesis, dilution, and degradation [39, 40]:

dPdt=α−μP−VmaxPKm+P

The synthesis rate, α, is dependent on physiological parameters related to cellular phenotype (e.g. growth rate, ribosomal availability, etc.) and to the plasmid construct (e.g. the protein, N-terminal region, RBS, promoter strength, plasmid copy number, mRNA stability, etc.) [41, 42]. Therefore synthesis rates cannot be compared between different target protein constructs (i.e., RFP synthesis may be different from Ntag-RFP). Dilution is represented with μ*P* term. Our balanced-growth experiments typically operate at around 10,000 target proteins per cell, as determined from correlation of activity versus abundance (**S2 Fig**). K_m_ values for ClpP-based degradation is around 600 proteins/cell[17]. We, accordingly, assume degradation to be zero order (*P* >> K_m_) and constant (*β)* under our balanced-growth experiments. Rearrangement of the differential equation yields

dPdt+μP=α−β

Thus, synthesis and degradation rates are equated to values that were measured. Specifically, protein and OD was measured over time. Rates (dP/dt and μ) could be calculated from the slopes at consecutive time points. *P* was the average protein between two time points. For a given biological replicate, at least three but no more than five time points were used to calculate α -β. Apparent degradation β was then calculated by subtraction from the no degradation control (Δ*clpP* or Δ*clpP* + pTrc). **S5 Fig** shows an example calculation of β from raw measurements.

For calculations and predictions of inducible time course dynamics, the full differential equation was used:

dPdt=α−μP−VmaxPKm+P

Based on purified mCherry and fluorescence measurements (**S2 Fig**), α = 40,000 synthesized proteins/cell h (unrepressed protein synthesis), Vmax = β = 15000 approximately 1 Fluorescence/OD600 = 10 proteins/cell. Specific parameters are proteins/cell h, K_m_ = 600 proteins/cell (approximately 1 μM), μ = 1 1/h, and P_0_ = 40,000 proteins/cell. Parameters were changed based on the tested condition (e.g. for synthesis repression only, V_max_ = 0 and α = 0). All numerical solutions were performed using the ode45 function on MATLAB R2014b (MathWorks, Natick, MA) running on a Macbook Pro running OS 10.9 (Apple, Cupertino, CA). All scripts are included in **S2 File** and all calculated data is included in **S5 File**.

## Results and Discussion

### Ntag is spontaneously cleaved *in vivo*

**Fig 2:**
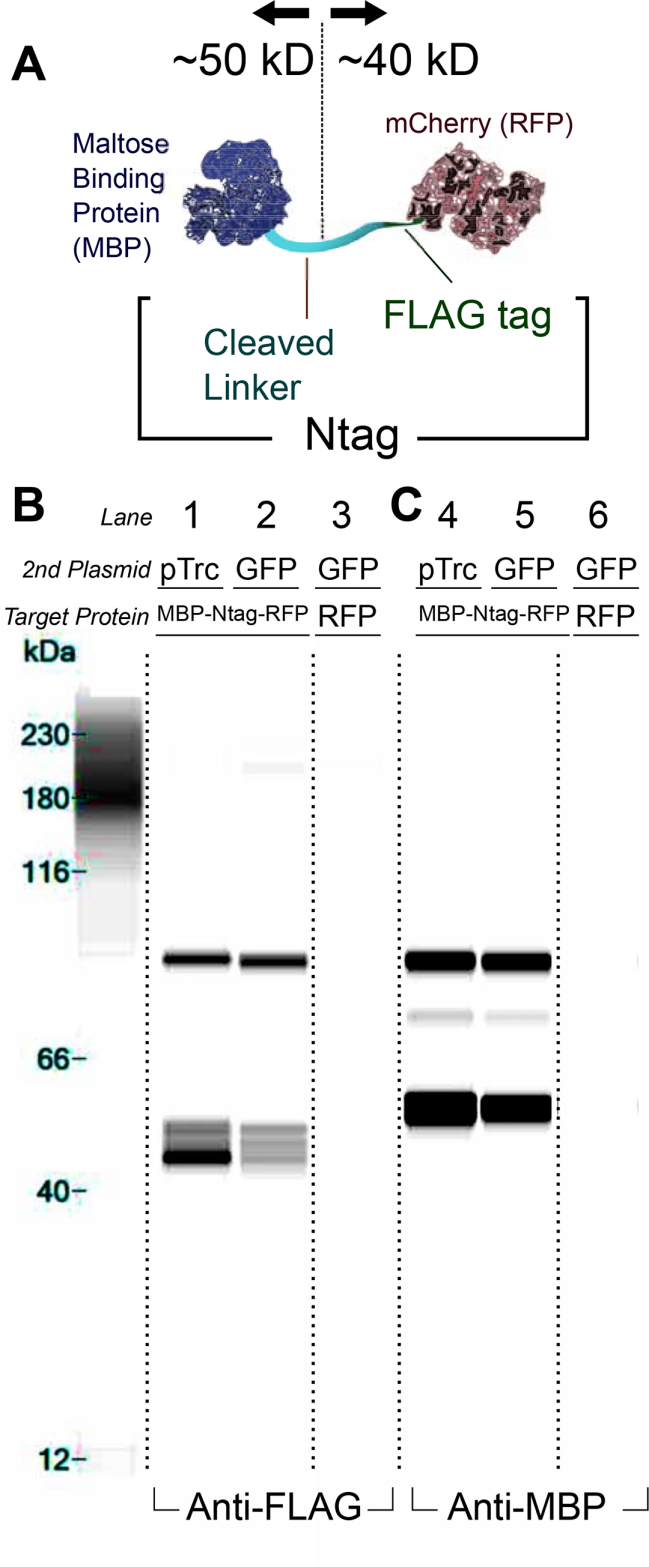
The Ntag is natively cleaved in *E*. *coli*. (**A**) A protein was designed to test for cleavage of the Ntag consisting of an N-terminal maltose binding protein (MBP) and a C-terminal RFP. The Ntag is equivalent to the linker sequence in the original TIPI study but with a FLAG epitope on the C-terminal end [27] (**B** and **C**). Cleavage was found in all conditions where the MBP-Ntag-RFP was expressed using immunoblotting techniques regardless of the expression of the perturbation plasmid (Lanes 1–2 and 4–5). A negative control for cellular background produced no relevant fragments (Lanes 3 and 6). The intact construct (~90kD) was observed when blotting with both an Anti-FLAG (**B**, Lanes 1–2) as well as Anti-MBP (**C**, Lanes 4–5) primary antibody. Consistent cleavage products were observed with the relevant antibodies: ~40 kD band with Anti-FLAG (Lanes 1–2) and ~50 kD band with Anti-MBP (Lanes 4–5). Band image was produced using the Compass software via a lane view option. Dotted lines indicate where original gel image is cropped for lanes not used in analysis. An unaccounted band at 70 kD is seen when immunoblotting with the Anti-MBP antibody (**C**, Lanes 4 and 5). This band is likely a C-terminally truncated MBP-Ntag-RFP construct. Sensitivity of the anti-MBP antibody is higher compared to anti-FLAG as suggested by the increased signal from the same 90 kD bands. Considering that the 70 kD bands only appears with anti-MBP, the abundance is likely at least an order of magnitude lower compared to the rest of the visualized products. Another spurious band is detected at around ~200 kD in Lane 2. This could be due to binding of the antibody to the lane standard. Protein Simple scientists have observed this phenomenon with many primary antibodies [43].

To characterize our system, we first evaluated cleavage of the Ntag protective domain and linker. To test cleavage in *E*. *coli*, we flanked the linker with two epitopes, MBP (N-terminal end of linker) and the FLAG tag (C-terminal end of linker / N-terminal end of mCherry). We denote the combined linker and FLAG tag region as the Ntag, which connected the upstream MBP to downstream RFP (**Fig 2A**). The cleaved product would be approximately 50 kD (MBP) and 40 kD (RFP). The full sequence of the Ntag is provided (**S1 File**).

First, we tested for proper cleavage of the target protein MBP-Ntag-RFP at the Ntag. Specifically, we measure cleavage under the context of two plasmids because of planned inducibility/perturbations described later in our study. We cotransformed two plasmids, a “target” plasmid and a “perturbation” plasmid. The target plasmid had high expression of MBP-Ntag-RFP (**Fig 2B**). The perturbation plasmid constitutively expressed either no protein (empty vector) or GFP (which was not expected to cleave MBP-Ntag-RFP) (Lanes 1–2 and 4–5 respectively). GFP expression was confirmed for all GFP conditions by measuring fluorescence (data not shown). Samples were taken in late stationary phase, and protein target cleavage was assayed by fragment size using an automated western blotter (The Wes). To test for antibody background with cellular lysate, we used a plasmid expressing untagged RFP in place of the MBP-Ntag-RFP protein (Lanes 3 and 6).

The MBP-Ntag-RFP construct is cleaved endogenously as shown when GFP or no protein is induced, therefore cleavage is independent of coexpressed proteins. This is evident by the ~40 kD anti-FLAG band that corresponds to RFP (**Fig 2B, Lane 1–2**) and a complementary ~50 kD anti-MBP band (**Fig 2C**, **Lanes 4–5**). Genomic MBP (*malE* gene) was knocked out to avoid background MBP staining (**Lanes 3** and **6**). We further tested growth phase-dependent and protease-dependent cleavage and found that cleavage occurs throughout the growth curve (**S6 Fig**).

We also confirmed that cleavage occurs at a single location on the linker. Despite multiple species at the Ntag-RFP portion (**Fig 2B, Lane 1–2**), only one band is seen for the MBP-Ntag portion (**Fig 2C** & **S6 Fig**). This observation suggests that cleavage occurs in one location and that the Ntag-RFP is processed proteolytically.

### Degradation occurs on different proteins fused with the Ntag

**Fig 3:**
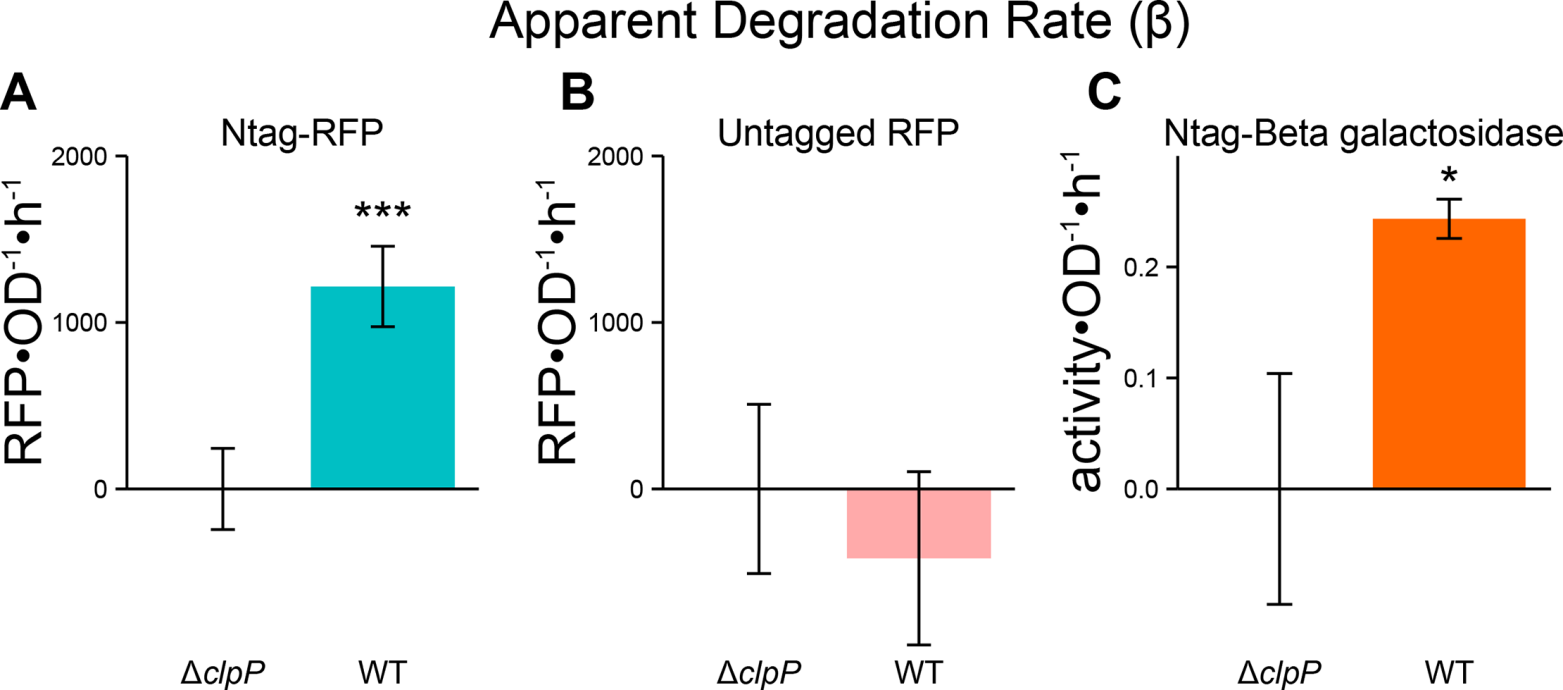
The Ntag confers ClpP-dependent degradation of RFP and beta-galactosidase. Measuring the apparent degradation rate (β) for the Ntag-RFP construct was significantly higher in the wildtype versus Δ*clpP* strain (**A**). For an untagged RFP, there is no appreciable degradation evident by the lack of apparent degradation rate (**B**). Fusing Ntag to beta-galactosidase resulted in similar degradation as measured by a significant β value (**C**). For all calculations of the apparent degradation rate, the respective Δ*clpP* condition is used to calculate the synthesis rate (α). This methodology is illustrated in **S5 Fig.** Single * designates p < 0.05 and triple (***) designates p < 0.001. All p values are calculated between ΔclpP and WT strains for given constructs. Values are mean ± s.e.m. (n = 4 to 10).

Spontaneous cleavage of the Ntag suggests the protein may be degraded through the N-end Rule mechanism present in bacteria [30]. We cultured cells expressing Ntag fused to RFP (Ntag-RFP) under balanced exponential growth, synthesis, and degradation conditions as described in **Methods** and measured growth rate and protein concentration per cell. Using the measured values, we calculated the apparent degradation rate (β) as illustrated in **S5 Fig.** The N-end Rule pathway in *E*. *coli* is known to require the ClpP protease for degradation of tagged substrate [30]. We, therefore, expect that for a putatively unstable protein with an N-degron, degradation rate β would be insignificant in a Δ*clpP* strain. We tested our Ntag-RFP construct in the Δ*clpP* strain versus wild type (WT) strain (strains described in **Table A in S1 File**). We see a significantly higher degradation rate in WT versus Δ*clpP* (**Fig 3A**). To verify that this is strictly a function of the Ntag, we show that with a stable mCherry construct (untagged RFP) that the degradation rate is very low and statistically indistinguishable from the Δ*clpP* strain with Ntag-RFP (**Fig 3B**). In these conditions, Δ*clpP* reduces growth rate by only 10%, indicating the deletion only has a minor effect on physiology (**S3 Fig**).

To verify that this degradation is related to the general presence of the Ntag and not an artifact of RFP, we fused Ntag to beta-galactosidase as well. We again see appreciable degradation for the Ntag-beta galactosidase construct suggesting that this tag engenders generalizable degradation (**Fig 3C**). Note: Under these conditions (high glucose, no IPTG/lactose), endogenous beta-galactosidate activity is undetectable (data not shown). We estimate that degradation rates between the RFP (~6×10^6^ residues•cell^-1^ •hr^-1^) and beta-galactosidase (~2×10^6^ residues•cell^-1^•hr^-1^) are consistent. Furthermore, we find that our calculated degradation rates compared well to established rates for native SsrA-tagged substrates, which are also ClpP dependent. For example, ArcA tagged with SsrA is approximately degraded at ~6.5×10^6^ residues•cell^-1^•hr^-1^ (full details in **S1 File**) [17, 44]. Raw values of RFP•OD^-1^ and OD over time are shown in **S5 Fig** to provide comparison with the model-derived calculations.

### Ntag-protein degradation is dependent on N-end rule pathway components

**Fig 4:**
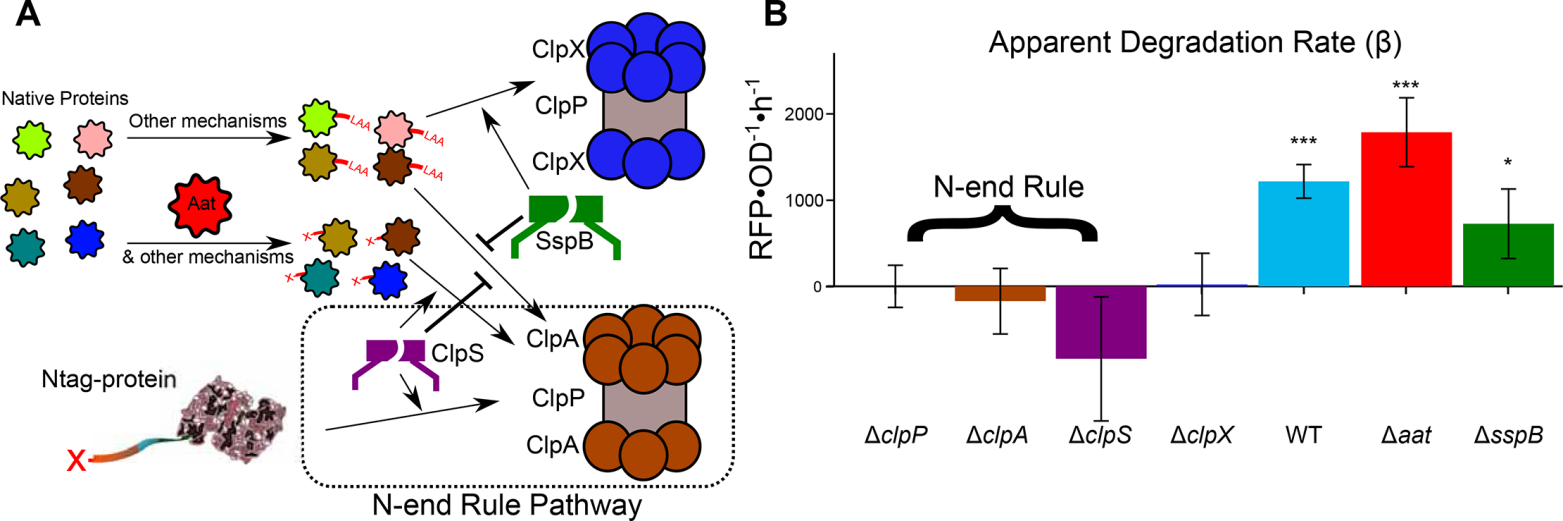
Ntag fused proteins are degraded via the N-end Rule pathway. The N-end Rule pathway requires an adaptor protein ClpS that promotes substrate toward the ClpAP protease complex (**A**). Native proteins are modified to have N-degrons via the Aat transferase enzyme or other mechanisms. These proteins with N-degrons are similarly facilitated by ClpS to the ClpAP complex for degradation as the Ntag fused target proteins. Another system tags native proteins with C-degrons, tags signaling degradation on the C-terminus. These proteins are preferentially degraded in the ClpXP complex by the SspB adaptor protein but can be degraded in the ClpAP complex. The apparent degradation rate (β) for the Ntag-RFP construct was measured across different knockouts of these degradation pathway members in comparison to the Δ*clpP* strain (**B**). The presence of N-end Rule members (ClpA, ClpS, ClpP) was required for degradation as indicated by no detectable degradation rate. The absence of ClpX reduced degradation to zero as well. Deletion of Aat resulted in, perhaps, enhanced degradation, and removal of the SspB adaptor protein reduced degradation but did not eliminate it. Single * designates p < 0.05 and triple (***) designates p < 0.001. Calculations of p-values are between Δ*clpP* and the given condition. Values are mean ± s.e.m. (n = 4 to 10).

N-terminal modification and degradation of our Ntag proteins by ClpP strongly suggests an N-end rule dependence mechanism. ClpA, ClpS, and ClpP are constituents of the *E*. *coli* N-end rule pathways (**Fig 4A)** [29, 30]. Native substrates for the terminal N-end rule protease, ClpAP, include proteins modified by the Aat transferase to reveal N-degrons [30, 32]. C-terminally-tagged proteins with the SsrA tag (AANDENYALAA) can also be degraded via the ClpAP protease [45]. Adaptor proteins ClpS and SspB are known to catalyze degradation by guiding substrate to protease complexes. Specifically, ClpS is known to facilitate degradation of proteins with N-degrons via ClpAP, and SspB is known to inhibit degradation of SsrA-tagged proteins via ClpAP but foster ClpXP-based degradation [28, 29, 46]. ClpXP is a structurally similar complex to ClpAP, and both use ClpP for protease activity; however, ClpXP has not been observed to degrade proteins with N-degrons [32].

To systematically determine which of the different pathway members in Ntag-based degradation are necessary, we measured apparent degradation rates for Ntag-RFP in strains with each pathway member described above knocked out (**Fig 4B**). In this study, growth rates changed by less than 20% and are accounted for in our analysis (**S3 Fig**). As expected, deletion of the other known N-end Rule components (Δ*clpA* and Δ*clpS*) abolished degradation. Interestingly, we see that Δ*clpX* unfoldase protein eliminates RFP degradation. We surmise that blocking the ClpXP pathway may result in saturation of the ClpAP pathway. In the absence of ClpX, both proteins with N-degrons or C-degrons would be processed by ClpAP. Under this postulation, the Ntag protein would not only be competing with internal proteins with N-degrons but would now also with, for example, proteins with a SsrA tag. Previously this saturation has been shown to decrease the apparent degradation rate of specific proteins due to a queuing effect, and therefore may be a possible explanation for no degradation under Δ*clpX* [39].

The auxiliary, degradation-related proteins, Aat and SspB only had modest effects on protein degradation. Δ*aat* should reduce the number of native proteins targeted for degradation, allowing more degradation capacity to be available for Ntag-RFP. We see a slight increase in degradation rate above WT. Δ*sspB* appears to reduce the degradation rate slightly, presumably by mitigating the ClpXP pathway, forcing more proteins to use the ClpAP pathway for degradation.

### Overexpressing some components of the N-end Rule pathway yields enhanced degradation

**Fig 5:**
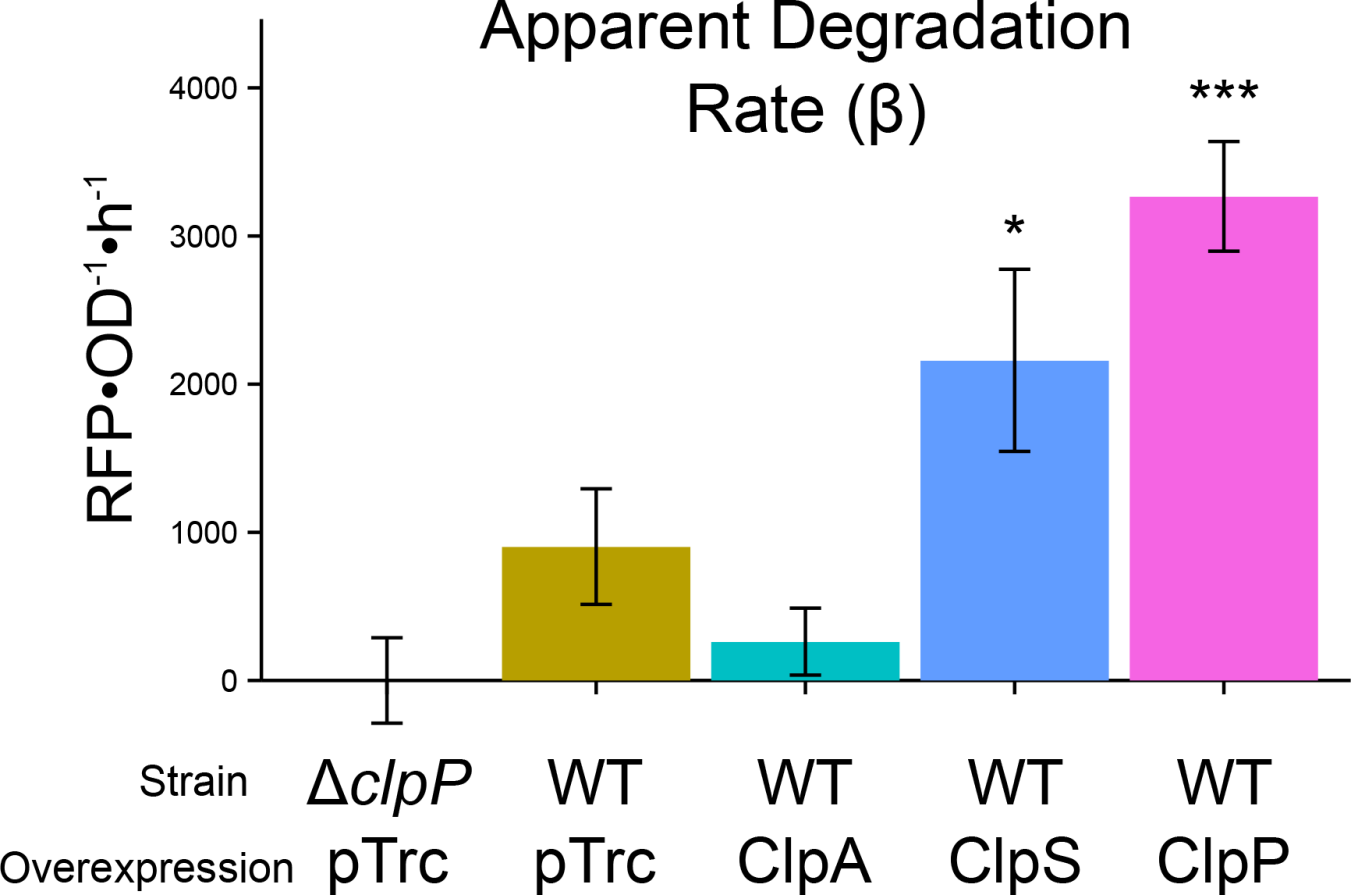
Ntag degradation is enhanced by ClpP and ClpS overexpression. Overexpression of a perturbation protein was used to test for degradation effects on Ntag-RFP. Overall degradation is reduced with the target/perturbation plasmid system as indicated by the control conditions (Δ*clpP* + pTrc versus WT + pTrc). ClpA overexpression did not change degradation detectably. Overexpression of each ClpP and ClpS enhanced the degradation rate. Single * designates p < 0.05 and triple (***) designates p < 0.001. Calculations of p-values are between Δ*clpP* + pTrc and the given condition. Values are mean ± s.e.m. (n = 4 to 6).

To improve the characteristic time of protein knockdown, protein degradation rates must be very high. We sought to identify the rate-limiting step from the N-end Rule pathway by overexpressing individual components and measuring β for Ntag-RFP **(Fig 5**). In all conditions, we verify growth rates within 10% (**S4 Fig**). In this experiment, the perturbation plasmid overexpresses a protein of interest under balanced growth conditions. Overexpression of ClpS and ClpP increase degradation, while other components did not. We note that ClpA overexpression may in fact yield less degradation compared to the wild-type control (WT + pTrc); however, we could not distinguish the conditions statistically. It is interesting that ClpA overexpression did not enhance degradation. ClpP overexpression resulted in the most dramatic increase in protein degradation rate, and is therefore the primary limiting step. Previous work has shown that ClpP may be the limiting stoichiometric component for formation of the ClpAP complex [47]. ClpS overexpression also improved degradation. ClpS’ enhancement role cannot be due to pathway limitation because only one is needed per ClpAP complex [48]. ClpS, however, is an inhibitor of other ClpA substrates such as SsrA tagged proteins [49]. ClpS overexpression, therefore, likely decreases substrate competition for Ntagged proteins.

We note that the control values of β are different than in the prior experiment, presumably due to introduction of the perturbation plasmid, IPTG addition, or antibiotics used to maintain the perturbation plasmid.

### Protein degradation is minor during rapid growth, but important in slow growth

**Fig 6:**
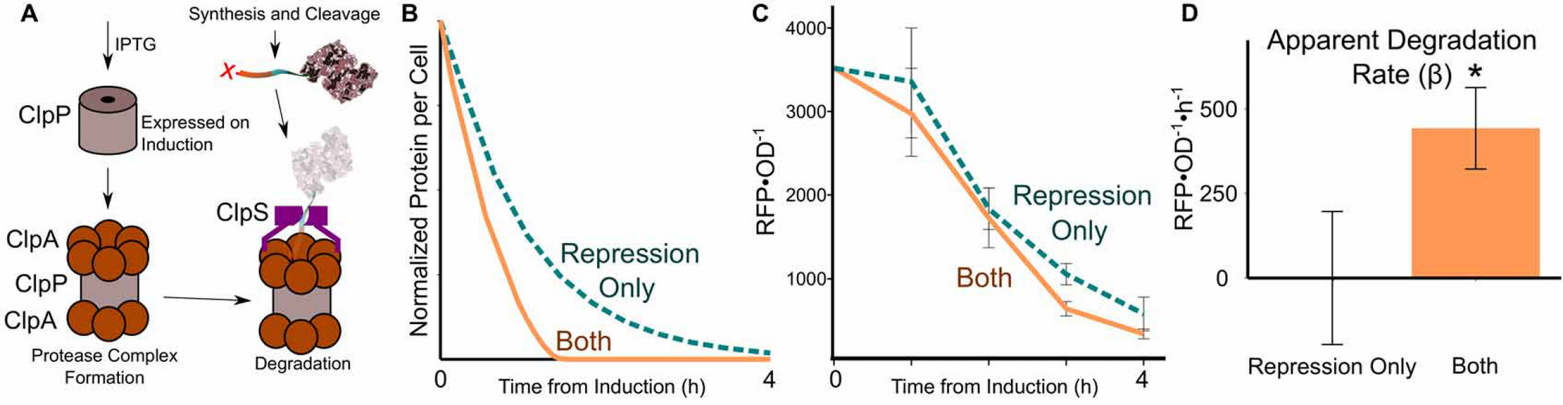
Degradation rate is minor for protein knockdown in rapidly growing cells. The target/perturbation plasmid system converts to an inducible, dynamic knockdown platform by temporally expressing ClpP within a *clpP* deletion strain (**A**). In high growth rate, knockdown is predicted to be primarily a result of dilution and degradation only slightly increases knockdown compared to synthesis repression alone (**B**). Experimental inducibility is tested using the target/perturbation system with Ntag-RFP as the target (**C**). β, degradation, increases post induction (**D**). Single * designates p < 0.05. All experimental values are mean ± s.e.m. (n = 6 to 8).

In the previous experiments, we measured degradation under a balanced growth state, however, the ultimate goal is to dynamically reduce protein for synthetic biology purposes. The two possible mechanisms are to repress synthesis (by TetR repression) or induce degradation. Because the presence or absence of ClpP results in large changes in degradation, the induction of ClpP should initiate the degradation of an Ntagged protein (**Fig 6A**). Furthermore, the presence or absence of ClpP was not significantly deleterious to fitness under balanced growth (**S3**and **S4 Figs**). Prior to conducting this experiment, we modeled protein concentration dynamics with parameters derived from our experiments (**Fig 6B**). Under maximal growth conditions (growth rates ~1.1 h^-1^), dilution effects are significant; therefore, synthesis repression is a valuable means for protein knockdown. Combined with degradation, slightly more knockdown is achieved, but the effect is minor during rapid growth as suggested by our modeling.

Consistent with the model, our measurements find that protein degradation only improves depletion slightly compared to repression alone (**Fig 6C**). However, the time required for knockdown is slower compared to our predictions (**Fig 6B**). This is likely due to a number of effects not considered in the model: (a) a delay associated with the required accumulation of ClpP or association with ClpA, (b) lag resulting from resuspending cells in fresh media at t = 0 and/or (c) leaky target protein expression due to residual doxycycline. A β estimate showed increased degradation post-induction (**Fig 6D**). The apparent β (~ 450 RFU•OD^-1^•h^-1^) in this experiment is lower than the β in the previous balanced growth experiments (1000–1800 RFU•OD^-1^•h^-1^). This lower rate may be due to lower concentration of the target protein (i.e., at the lower concentration, degradation may be a first order rate). Ntag-RFP concentration was lower in the induction experiments (100–3500 RFU•OD^-1^) compared to balanced growth where there was no repression (1000–20000 RFU•OD^-1^).

Looking forward, we expect induced protein degradation to be particularly valuable for slowed growth conditions where dilution effects are small. In general, we see the knockdowns efficiencies improve for combining degradation and synthesis repression versus simply synthesis repression as growth rates are lowered. Slow growth conditions are particularly pertinent when essential enzymes are the targets of protein degradation. The less of the essential enzyme, the slower the growth, as highlighted in this review [16].

In this study, we did not consider how degradation rate is affected by the sequence or structural features of the Ntag. Previous studies have found specific features of N-degrons that affect degradation rate. For example, acidic residues adjacent to the N-terminus (after cleavage) reduce degradation rates [50], while aliphatic residues increase the degradation rate [51]. A separate study was able to tune the degradation rate through modification of the ClpS adaptor protein [52]. These structural features could be used to rationally design an enhanced Ntag degradation system.

We have expanded the options for inducible protein degradation by establishing the Ntag system for facile, generalizable degradation of proteins by using a genetically encoded N-terminal modification. We implemented a modeling framework that focused on degradation rates, which allowed systematic analysis of the effects of protein degradation pathway components. We verify Ntag generalizability, N-end Rule dependence, enhancement conditions, and an inducible strategy. Our tool should be useful to a variety of metabolic engineering applications, protein-based circuits, and study of essential proteins.

## Supporting Information

S1 Fig Titrating doxycycline allows controllable synthesis of target protein. Untagged RFP is driven by expression from a pl-tetO promoter. In MG1655 Z1 strain, constitutive expression of TetR represses synthesis of RFP. Synthesis can be controlled by adding varying levels of doxycycline, which is known to sequester TetR. Measurements were performed at a working volume of 200 μL in a 96 well plate running in Synergy H1 Plate Reader (Biotek, Winooski, VT).
(EPS)

S2 Fig Purification of MBP-Ntag-RFP (from pKS011) allows for a rough correlation between fluorescence and protein number. Method for protein purification can be found in **S1 File.**
(EPS)

S3 Fig Growth rates for balanced growth one plasmid experiments. Raw data available in **S3 File**.
(EPS)

S4 Fig Growth rates for balanced growth two plasmid experiments. Raw data available in **S4 File**.
(EPS)

S5 Fig An overview of apparent degradation calculation. Raw protein abundance/activity and biomass (OD) are measured during balanced growth (exponential phase) (**1**). From the data, dP/dt, μ, and P are calculated and used to calculate α-β over time (**2** and **3**). Average α-β is computed (**4**) and then apparent degradation (β) yields from the subtraction of the wild type condition from the control condition (in this case, Δ*clpP*) (**5**). Note: this data is from one day of experiments and thus does not correspond to **Fig 3**(n = 2). Raw data for this figure is available in **S7 File**.
(EPS)

S6 Fig Time-course assay shows one cleavage event and cleavage occurring throughout the growth curve. In this assay, MBP-Ntag-RFP was coexpressed with a second plasmid either the empty pTrc plasmid (P) or pJB028 (28). Cells were grown to midlog phase (OD = 0.6) at t0, at which point a sampling occurred (**Lanes 1 and 2**). IPTG was then added to one of the conditions (28 + I) and sampled separately (**Lanes 5** and **8**). Sampling occurred again 1 (**Lanes 3**, **4, and 5**) and 3 (**Lanes 6, 7**, **and 8**) hours later. Intact MBP-Ntag-RFP is seen throughout with Anti-MBP antibody as indicated by band at around 85 kDa. Cleavage products seen throughout as indicated by 45 kD band on Anti-MBP blot and 40 kD bands on Anti-FLAG. Furthermore, a single cleavage event is indicated suggested by a singular band for the Ntag-MBP portion.
(EPS)

S1 File Supplemental Information. This document includes supplementary tables, molecular cloning information (sequences, oligonucleotides), methods for protein purification, and calculations of protein degradation rates.
(PDF)

S2 File Plasmid GenBank files and MATLAB code. GenBank files for all plasmids used in this study and MATLAB code for protein knockdown simulation.
(ZIP)

S3 File Raw Data for Single Plasmid Experiments. Excel file with all measurements of OD, fluorescence, beta-galactosidase activity, and calculations for apparent degradation rate.
(XLSX)

S4 File Raw Data for Double Plasmid Experiments. Excel file with all measurements of OD, fluorescence, and calculations for apparent degradation rate.
(XLSX)

S5 File Knockdown simulation data. Data used in **Fig 6**from MATLAB simulation.
(CSV)

S6 File Raw Data for Induction Experiments. Excel file with all measurements of OD, fluorescence and calculations for the induction experiments.
(XLSX)

S7 File Raw Data and Calculation for S5 Fig. Excel file with all measurements of OD, fluorescence and calculations for **S5 Fig** calculations.
(XLSX)
