# Supplementary material for: N-Terminal-Based Targeted, Inducible Protein Degradation in Escherichia coli
Source: PLoS One. 2016 Feb 22;11(2):e0149746. doi: 10.1371/journal.pone.0149746 (PMC4765774; doi:10.1371/journal.pone.0149746)
Supplement: S1 File — This document includes supplementary tables, molecular cloning information (sequences, oligonucleotides), methods for protein purification, and calculations of protein degradation rates. (PDF) [file pone.0149746.s007.pdf]

S1 File - Supplementary Information

for "N-terminal-based targeted, inducible protein degradation in Escherichia coli"

Sekar, K., Gentile, A.M., Bostick, J.W., and Tyo, K.E.J.

Contents

1 Molecular Cloning Information **2**
1.1 Plasmids 2
1.2 Ntag Protein Sequence 2

2 Plasmid, Strain, and Construction Information **3**

3 Supplemental Methods **7**
3.1 Purification of MBP-Ntag-RFP 7
3.2 Methods for Time Course Westerns 7

4 Back of the envelope calculations for degradation rates **8**
4.1 Ntag-mCherry calculation 8
4.2 Ntag-beta-galactosidase calculation 8
4.3 arcA-ssrA calculation 9

1 Molecular Cloning Information

1.1 Plasmids

All plasmids GenBank files are included separately in ZIP file. Plasmid kits available from AddGene.

1.2 Ntag Protein Sequence

MSITSLYKKAGSENLYFQFHKSGAWKLPVSLVKRGIDKLDYKEQLQAWRWEREIDERN RPLSDEELDAMFPEGYKVLPPPAGYVPIRTPAHMDRIPAARTTENSSDYKDDDDK ... (start of protein)

Features:
FLAG tag in green
Linker in black

2 Plasmid, Strain, and Construction Information

Table A: Strains used in this study.

Name	Genotype	Resistance	Purpose
MG1655 Z1	F-, lambda-, rph-1, laciq, PN25-tetR, SpR	(none)	E. coli MG1655 Z1 host that produces constitutive levels of TetR, LacI and AraC from the chromosome
MG1655 Z1 malE	F-, lambda-, rph-1, laciq, PN25-tetR, SpR, malE-	(none)	E. coli MG1655 Z1 host that produces constitutive levels of TetR, LacI and AraC from the chromosome with additional malE KO
MG1655 Z1 malE clpP	F-, lambda-, rph-1, laciq, PN25-tetR, SpR, malE-, clpP-	(none)	E. coli MG1655 Z1 host that produces constitutive levels of TetR, LacI and AraC from the chromosome with additional malE, clpP KO
MG1655 Z1 malE aat	F-, lambda-, rph-1, laciq, PN25-tetR, SpR, malE-, aat-	(none)	E. coli MG1655 Z1 host that produces constitutive levels of TetR, LacI and AraC from the chromosome with additional malE, aat KO
MG1655 Z1 malE clpA	F-, lambda-, rph-1, laciq, PN25-tetR, SpR, malE-, clpA-	(none)	E. coli MG1655 Z1 host that produces constitutive levels of TetR, LacI and AraC from the chromosome with additional malE, clpA KO
MG1655 Z1 malE clpS	F-, lambda-, rph-1, laciq, PN25-tetR, SpR, malE-, clpS-	(none)	E. coli MG1655 Z1 host that produces constitutive levels of TetR, LacI and AraC from the chromosome with additional malE, clpS KO
MG1655 Z1 malE clpX	F-, lambda-, rph-1, laciq, PN25-tetR, SpR, malE-, clpX-	(none)	E. coli MG1655 Z1 host that produces constitutive levels of TetR, LacI and AraC from the chromosome with additional malE, clpX KO
MG1655 Z1 malE sspB	F-, lambda-, rph-1, laciq, PN25-tetR, SpR, malE-, sspB-	(none)	E. coli MG1655 Z1 host that produces constitutive levels of TetR, LacI and AraC from the chromosome with additional malE, sspB KO

Table B: Plasmids used in this study.

Short Name and Insert	Plasmid Name	Description	Backbone	Antibiotic	Promoter
MBP-Ntag-RFP	pKS011	Construct made for cleavage experiments.	pSB4C5 (pSC101)	Cm (low)	PlTetO
Ntag-RFP	pKS012	Testing Ntag degradation on mCherry	pSB4C5 (pSC101)	Cm (low)	PlTetO
RFP	pKS013	Negative control mCherry	pSB4C5 (pSC101)	Cm (low)	PlTetO
Ntag-beta galactosidase	pKS014		pSB4C5 (pSC101)	Cm (low)	PlTetO
pTrc	pTrc	Empty pTrcHis2B plasmid	n/a	Amp	Trc
GFP	pJB005	pTrc plasmid expressing GFP on IPTG addition	ptrc	Amp	Trc
TEV	pJB028	pTrc plasmid expressing p14*-TEV on IPTG addition	ptrc	Amp	Trc
clpA	pKS042	pTrc plasmid expressing clpA on IPTG addition	ptrc	Amp	Trc
clpS	pKS043	pTrc plasmid expressing clpS on IPTG addition	ptrc	Amp	Trc
clpP	pKS044	pTrc plasmid expressing clpP on IPTG addition	ptrc	Amp	Trc

Table C: Construction Oligonucleotides

Primer Name	Purpose	5' to 3' sequence	Template
KS139	Constructing pKS011	GCTGTACAAGTAATAAATATCCGGGTAGGCGCAA	Unpublished pSB4C5 (pSC101) plasmid
KS140	Constructing pKS011	CTGTTTTTATTTTCATGCGGTACCTTTCTCCTCT	Unpublished pSB4C5 (pSC101) plasmid
KS141	Constructing pKS011	GGAGAAAGGTACCGCATGAAAATAAAAACAGGTGCACG	pMAL-p5X (from NEB)
KS142	Constructing pKS011	CGTGATCGACCCGAGGTTGTTGTTATTG	pMAL-p5X (from NEB)
KS143	Constructing pKS011	GACGATGACAAGATGGTGAGCAAGGGCGAG	gBlock synthesized from IDT
KS144	Constructing pKS011	CGCCTACCCGGATATTTATTACTTGTACAGCTCGTCCATG	gBlock synthesized from IDT
KS145	Constructing pKS011	CAACCTCGGGTCGATCAC	Unpublished mCherry plasmid
KS146	Constructing pKS011	CCTTGCTCACCATCTTGTC	Unpublished mCherry plasmid
KS147	Constructing pKS012	CGACATGCGGTACCTTTCTCCTCT	pKS011
KS148	Constructing pKS012	AAGGTACCGCATGTCGATCACGTCGCTGTAT	pKS011
KS073	Constructing pKS013	CCTTGCTCACCATGCGGTACCTTTCTCCTCT	pKS012
KS074	Constructing pKS013	AGAAAGGTACCGCATGGTGAGCAAGGGCGAG	pKS012
KS149	Constructing pKS014	TGGTCTGGTGTCAAAAATAAATATCCGGGTAGGCGCAATCACTTTCG	pKS012
KS150	Constructing pKS014	TCCGTAATCATGGTCATCTTGTCATCGTCATCTTTGTAGTCGC	pKS012
KS151	Constructing pKS014	TGACGATGACAAGATGACCATGATTACGGATTCACTGG	E. coli Genome
KS152	Constructing pKS014	TGCGCCTACCCGGATATTTATTTTTGACACCAGACCAACTG	E. coli Genome
KS230	Putting clp proteins into ptrc (inverse PCR)	AGAGCTCGAGATCTGCAGCTGG	pTrcHisB
KS231	Putting clp proteins into ptrc (inverse PCR)	CATCCTCTGGATCCATGGTTTATTCCTCC	pTrcHisB
KS232	Making pKS042 (clpA)	GGAGGAATAAACCATGGATCCAGAGGATGCTCAATCAAGAACTGG	E. coli Genome
KS233	Making pKS042 (clpA)	CCAGCTGCAGATCTCGAGCTCTTAATGCGCTGCTTCCGCC	E. coli Genome
KS234	Making pKS043 (clpS)	GGAGGAATAAACCATGGATCCAGAGGATGGGTAAAACGAACGACTGGCTGG	E. coli Genome
KS235	Making pKS043 (clpS)	CCAGCTGCAGATCTCGAGCTCTCAGGCTTTTTCTAGCGTACACAGC	E. coli Genome
KS236	Making pKS044 (clpP)	GGAGGAATAAACCATGGATCCAGAGGATGTCATACAGCGGCGAAC	E. coli Genome
KS237	Making pKS044 (clpP)	CCAGCTGCAGATCTCGAGCTCTCAATTACGATGGGTCAGAATCGAATCG	E. coli Genome
JB045	Making pJB005 - Digest and Gibson into pTrc	GACCGAATTCATTAAAGAGGAGAAAGGTACCATGCGTAAAGGAGAAGAACTTTTCACTGGAG	pZE-GFP construct
JB046	Making pJB005 - Digest and Gibson into pTrc	CCTGCAGCTGCTCTTTGTATAGTTCATCCATGCCATGTGTAATCCC	pZE-GFP construct
JB043	Making pJB028 ancestor - Digest and Gibson into pTrc	CGATTAAATAAGGAGGAATAAACCATGGATCCAGAGGATGGCAATGCAGGCAGCAAAACG	Synthesized Sequence
JB044	Making pJB028 ancestor - Digest and Gibson into pTrc	CCATATGGTACCAGCTGCAGATCTCGAGCTCTTACAGCTGGGTTGCTTCTTTAACCGGC	Synthesized Sequence

Table D: Checking Oligonucleotides

Primer Name	Purpose	5' to 3' sequence
JB051 (s)	TEV Chk	GTAGCGCAATGGGTGAAAGCCTG
JB052 (a)	TEV Chk	CTGGGTTGCTTCTTTAACCGGCTG
JB053 (s)	GFP Chk	AAGAGTGCCATGCCCGAAGG
JB054 (a)	GFP Chk	CCTTCGATTCCGACCTCATTAAGCAGC
KS076 (a)	checking for mCherry	CATGTTATCCTCCTCGCCCTT
KS077 (s)	checking for mChery	CCATCGTGGAACAGTACGAAC
KS131(s)	checking for MBP	GTTAATAAAGACAAACCGCTGGGTGC
KS132 (a)	checking for MBP	GCAACCTGTGGGAATTTCTCTTCC
KS055 (s)	checking for lacZ	AAGGCACATGGCTGAATATCGAC
KS022 (a)	checking for lacZ	ATGCGCTCAGGTCAAATTCAG
JB071 (s)	Target plasmid check	GGCGTATCACGAGGCCCTTTC
KS056 (a)	Target plasmid check	TCCTACTCAGGAGAGCGTTCAC
JB011 (s)	pTrc plasmid check	TGTGGGCACTCGACCGGAATTATC
KS191(a)	pTrc plasmid check	GGCGACACGGAAATGTTGAATAC
KS186 (a)	clpX checking	CCCTGCGGTCAGCAAGAAAG
KS187 (s)	clpP checking	CTGATCAAGTTTGGTCTTATCCCTGAG
KS189 (s)	clpA checking	ATAACAGCACCGATGCGATG

3 Supplemental Methods

3.1 Purification of MBP-Ntag-RFP

E. coli MG1655 K12 $\Delta malE$ was transformed with pKS011. An individual colony was picked and cultured in 50 mL in same conditions as described in Methods. The next day, the culture was chilled and kept cold throughout the consequent steps. The culture was homogenized using an EmulsiFlex-C5 homogenizer (Avestin, Ottawa, Canada) and settled for 40 minutes at 18,000g to remove cellular debris. MBP-Ntag-RFP was purified from supernatant using the ÄKTAxpress Twin FPLC system (General Electric, Fairfield, Connecticut) using the MBPTrap HP column (GE Healthcare Life Sciences, Fairfield, Connecticut). Purification protocol provided with column was used to perform purification. Protein was quantified using spectrophotometer and standards were prepared in the elution buffer.

3.2 Methods for Time Course Westerns

E. coli MG1655 K12 $\Delta malE$ were chemically transformed with pKS011 and pTrc or pJB028 (p14*-TEV) perturbation plasmids. Colonies were grown on plates with respective antibiotics and used to seed 5mL LB at 37°C and 250RPM for overnight culture. Cells were subcultured to 0.05 OD_{600} and allowed to grow until 0.6 OD_{600}, upon which 1mM IPTG was added for the designated conditions. Cells were harvested before IPTG addition (t = 0h), after 1 hour, and after 3 hours. Cells were pelleted, resuspended in 500μL ice cold PBS with 2.5μL protease inhibitor cocktail (Sigma) and probe-tipped sonicated. Protein concentration was determined by Bradford assay (Biorad, Berkeley, CA) according to supplied protocol. 20 μg protein was loaded into each well of an AnyKD polyacrylamide gel (Biorad, Berkeley, CA), electrophoretically separated, and transferred to PVDF (EMD Millipore, Billerica, MA) using Towbin's method. Membranes were blocked with 10% nonfat dry milk (Nestle USA, Washington, DC) in TBS-T and washed three times in a large volume of TBS-T for 10 minutes with gentle agitation. Proteins were visualized using a monoclonal mouse anti-FLAG primary antibody (Antibody Registry#: AB_262044, Product No. F1804, Sigma Aldrich, St. Louis, MO) at a dilution of 1:1500. The anti-mouse HRP secondary antibodies (Cell Signaling Technology, Danvers, MA) at a dilution of 1:30000 was used for the next incubation. And finally the bands were visualized using the WesternC chemiluminescent substrate (Biorad, Berkeley, CA) on a ChemiDoc XRS+ (Biorad, Berkeley, CA). The experiment was also performed using a polyclonal rabbit anti-MBP primary antibody (Antibody Registry#: AB_675709, Product No. sc-808, Santa Cruz Biotech, Santa Cruz, CA) on the same protein lysates at a 1:1500 concentration.

Anti-rabbit secondaries were used at the same concentration (1:30000). Dilutions were determined from Western optimization.

4 Back of the envelope calculations for degradation rates

4.1 Ntag-mCherry calculation

Numbers and assumptions:

- β (from single plasmid experiments) - 1500 Fluorescence/OD hr
- conversion of Fluorescence/OD to protein/cell -10 (proteins/cell)/(fluorescence/OD) (from purified RFP standard curve)
- Residues in Ntag-mCherry - 350 residues/protein

$$(1500)(10)(350) \approx 5 \times 10^6 \frac{\text{residues}}{\text{cell hr}} \tag{1}$$

4.2 Ntag-beta-galactosidase calculation

Numbers and assumptions:

- β (from single plasmid experiments) - 0.2 activity/mL hr OD
- activity to protein mass - 0.002 (mg/activity) (based on Bulletin from Sigma)
- Beta-gal molecular weight - 7.7×10^{-6} mol/g
- OD to cells/mL - 1×10^{-9} OD/(cells/mL)
- Residues in Ntag-Beta gal - 1137 residues/protein

$$(0.2)(0.002)(0.001\frac{\text{g}}{\text{mg}})(7.7\times10^{-6})(1\times10^{-9})(6.02\times10^{23}\frac{\text{proteins}}{\text{mol}})(1137) \approx 2\times10^6 \frac{\text{residues}}{\text{cell hr}} \tag{2}$$

4.3 arcA-ssrA calculation

Numbers and assumptions:

- Rough number of $clpX_6$ in a cell according to Farrell et al. - 90 proteins/cell
- Vmax for ArcA with ssrA tag from McGinness et al. - 5 1/(min $clpX_6$) (Note: from *in vitro* experiments)
- Residues in ArcA w/ ssrA - 240 residues/protein

$$(90)(5)(60\frac{\text{min}}{\text{hr}})(240) \approx 6.5 \times 10^6 \frac{\text{residues}}{\text{cell hr}} \tag{3}$$
